# Compartment‐Specific Variation in Bacterial Microbiome and Polyphyllin Profiles in *Paris polyphylla*

**DOI:** 10.1155/ijm/1725012

**Published:** 2026-07-08

**Authors:** Xinhong Wu, Yan Deng, Shihui Li, Kai Zou, Zhenchun Duan, Nazidi Ibrahim, Jin Zhou, Luhua Jiang, Xueduan Liu, Shaodong Fu, Yili Liang

**Affiliations:** ^1^ School of Resource Processing and Bioengineering, Key Laboratory of Biometallurgy of Ministry of Education, Central South University, Changsha, China, csu.edu.cn; ^2^ Hunan Institute of Microbiology, Hunan Academy of Agricultural Sciences, Changsha, China, hnbemc.com; ^3^ College of Advanced Materials Engineering, Jiaxing Nanhu University, Jiaxing, Zhejiang, China

**Keywords:** ecological niche differentiation, interactions, microbiome, *Paris polyphylla*, polyphyllin

## Abstract

*Paris polyphylla* (*P. polyphylla*) is a valuable traditional Chinese medicinal plant, yet the spatial distribution of its compartment‐specific bacterial microbiomes and their correlative relationships with bioactive polyphyllins remain poorly characterized. Here, we combined 16S rRNA amplicon sequencing, metabolite analysis, and bioinformatics to investigate the distribution patterns of bacterial communities and polyphyllins across bulk soil (BS), rhizosphere soil (RS), root endospheres (REs), stem endospheres (SEs), and leaf endospheres (LEs) of *P. polyphylla*. A spot inoculation assay was further used to verify the interactions between the dominant genus *Pseudomonas* (strain *Pseudomonas palleroniana* P6) and key polyphyllin I and VII. The results showed that polyphyllin I and II were highly accumulated in aerial SEs and leaves, whereas polyphyllin VI, VII, and diosgenin were predominantly concentrated in REs. Bacterial diversity and richness showed a gradual decline from BS to LE, with ecological niche differentiation identified as the primary driver of bacterial community divergence across compartments, which was further modulated by polyphyllin content. *Pseudomonas*, the dominant genus in all compartments, displayed a decreasing relative abundance with ascending compartmental niches, and its abundance was significantly negatively correlated with polyphyllin I levels but positively correlated with polyphyllin VII levels—a trend experimentally validated by gradient polyphyllin concentration‐based microbial growth assays. Redundancy analysis (RDA) indicated that polyphyllin content (especially VI, VII, and diosgenin) significantly influenced bacterial community composition. Additionally, *P. polyphylla* exhibited selective enrichment of beneficial microbes, with selection pressure intensifying progressively across compartments. This study clarifies the compartment‐specific distribution patterns of bacterial microbiomes and polyphyllins in *P. polyphylla* and their correlative relationships, deepens the understanding of plant‐microbiome interactions in medicinal plants, and provides a theoretical basis for optimizing *P. polyphylla* cultivation strategies and developing microbial inoculants for sustainable agricultural production.

## 1. Introduction

There are various microorganisms that parasitize or coexist inside and outside plants. These microbes can promote plant growth, resist pathogen invasion, and even metabolize various bioactive substances such as medicinal ingredients [1–3]. Many factors affect the effective ingredient content of medicinal plants, such as soil properties (pH, salinity, and heavy metal concentration), gas composition (CO_2_/O_2_ levels), seasonal changes (temperature, drought, and light), planting duration, plant varieties, and differences in plant compartments, which can all affect the efficacy of medicinal plants [4–7]. Similarly, these factors can also affect microorganisms that coexist or parasitize with medicinal plants [8]. Previous research has shown that microorganisms can affect the effective ingredients of medicinal plants by regulating host metabolism and even participating in the synthesis pathway of active ingredients [9]. However, different plant compartments exhibit distinct selectivity and suitability for microorganisms, leading to heterogeneous distribution of microbes within and outside the plant [10–12]. Conversely, plant metabolic activities also influence the survival status and activity of microorganisms, for example, plant rhizosphere secretions modulate the structure and function of rhizosphere microbial communities [13–15]. The differences in plant compartments and metabolism significantly impact microbial distribution and function [11, 16]. Studying the ecological niche distribution patterns of microorganisms in different compartments of plants can enhance our understanding of the complexity of plant‐microbial interactions [7].

Ecological niche differentiation refers to the process by which coexisting microbial taxa partition environmental resources or habitats, thereby reducing competition and enabling stable coexistence [3, 17]. In the context of plant microbiomes, different plant compartments—such as the rhizosphere soil (RS), root endosphere (RE), stem endosphere (SE), and leaf endosphere (LE)—represent distinct ecological niches that impose unique selective pressures on microbial communities [18, 19]. These niches vary in physical structure, nutrient availability, oxygen tension, and host immune surveillance, collectively driving the spatial heterogeneity of microbial assemblages along the soil–plant continuum [20, 21]. Previous studies on *Populus*, *maize*, and *Arabidopsis* have demonstrated that compartment niche is a primary determinant of microbial community assembly, with pronounced declines in diversity and shifts in community composition from belowground to aboveground tissues [22–24]. However, how compartment‐specific niche differentiation shapes the bacterial microbiome and its interaction with bioactive polyphyllins in the medicinal plant *Paris polyphylla* (*P. polyphylla*) remains poorly understood.

The primary bioactive compounds isolated from *P. polyphylla* are polyphyllin I, II, VI, and VII, which exhibit antibacterial, antiviral, antioxidant, and antitumor properties, establishing *P. polyphylla* as a valuable medicinal resource in traditional Chinese medicine [25, 26]. Consequently, the market demand for high‐quality *P. polyphylla* with elevated polyphyllins content is substantial [27]. Plant‐microbe interactions, which regulate secondary metabolism, remain a key research focus in this context [28]. Current studies on *P. polyphyll*‐associated microorganisms have primarily employed isolation, cultivation, and 16S rRNA amplicon sequencing to characterize endophytic communities. For instance, Li et al. [29] isolated 63 endophytic fungi from *P. polyphylla* var. *yunnanensis* rhizomes, predominantly belonging to *Fusarium* and *Aspergillus*. Subsequent studies revealed significant variations in fungal community composition across different plant compartments and altitudinal gradients, and demonstrated that fungal diversity fluctuates with plant age [30–32]. However, these investigations have primarily focused on community composition and diversity, with limited attention given to the functional relationships between these microbes and the host′s secondary metabolites [29–32]. Emerging evidence from other medicinal plants suggests that plant‐associated microbes can influence the accumulation of bioactive compounds by modulating host metabolism or participating in biosynthetic pathways [9, 16]. For example, endophytic bacteria affect flavonoid content in *Ginkgo biloba* leaves [9], and rhizosphere microbes regulate steroidal saponin production in various medicinal plants [28, 33]. In *P. polyphylla*, a few studies have hinted at potential links between microbial communities and polyphyllin accumulation [31, 34], but systematic investigations into how compartment‐specific bacterial microbiomes interact with polyphyllin biosynthesis remain scarce. Specifically, the bidirectional relationships—how bacterial communities shape polyphyllin profiles and how polyphyllins, in turn, influence bacterial colonization and assembly across different plant niches—have not been experimentally addressed. Therefore, elucidating these complex interactions is essential for understanding the ecological and functional roles of the *P. polyphylla* microbiome and for developing microbiome‐based strategies to enhance polyphyllin production.

The relationship between bacterial communities and polyphyllin content in *P. polyphylla* is poorly characterized, necessitating investigation into ecological niche differentiation of the bacterial communities and its driving factors. Elucidating compartment‐specific interactions between bacterial communities and polyphyllin is critical advancing sustainable cultivation strategies of this high‐value medicinal plant. This study investigates RS, roots, stems, and leaves of *P. polyphylla* with different growth years. Using 16S rRNA amplicon sequencing, liquid chromatography‐mass spectrometry‐based metabolomics, and bioinformatics, we elucidated bacterial community composition and recruitment mechanism different compartments. Additionally, we analyzed the ecological niche spatial distribution patterns of bacterial communities and their interaction with the types and contents of polyphyllin, providing a theoretical foundation for optimizing cultivation practices and enhancing bioactive compound yields in *P. polyphylla*.

## 2. Materials and Methods

### 2.1. Sample Collection and Pretreatment

The *P. polyphylla* samples were collected from the Jiudingshan area of Dali City, Yunnan Province, China. The detailed location was 25°20 ^′^24 ^″^ N, 100°29 ^′^18 ^″^ E with an altitude of 1700–3100 m. Jiudingshan experiences a climate that is marked by humidity and warmth, featuring an average yearly temperature of 17.3°C, a mean annual rainfall of 824 mm, and an average of 2339.5 h of sunshine annually. Seeds from closely related maternal plants of the same ancestry were sown in a 100 × 20 m greenhouse in May 2014. Consistent water and fertilizer management practices have been applied annually, including regular and quantified watering without additional top dressing. During the initial 3 years, the *P. polyphylla* plants were relatively small, presenting challenging to obtain sufficient biomass for sample testing. Consequently, this study focused on plants collected in September 2018, 2019, and 2020, representing 4‐year‐old, 5‐year‐old, and 6‐year‐old specimens, respectively. Three 3 × 3 m quadrats were established, and 15 *P. polyphylla* plants of similar size were randomly selected from each quadrat using a 5‐point sampling method. Samples were collected from the bulk soils (BSs), RSs, RE, SE, and LE of *P. polyphylla* plants grown for 4, 5, and 6 years, respectively. These samples were labeled as 4, 5, and 6 (e.g., BS4, BS5, and BS6). For BS samples, soil was collected 5 cm away from the *P. polyphylla* roots at a depth of 5–15 cm. After removing loose soil, the roots with adhering soil were treated according to Saunders et al.′s [35] method, and precipitate was collected as RSs. The surface disinfection of roots, stems, and leaves follows the method of Fu et al. [36]. After surface sterilization, the roots, stems, and leaves were dried on a sterile workbench. The water from the final rinse was inoculated onto a culture medium. The absence of growth on the culture medium after incubation for 7 days at 30°C confirmed successful surface disinfection. Although validation through the absence of culturable growth from the final rinse water cannot completely exclude potential contributions from residual extracellular DNA, DNA from dead or nonculturable cells, or material from tightly adherent biofilms to the endophytic sequencing libraries [37, 38], which remains a widely accepted standard practice in the field [39, 40]. Therefore, the separation between epiphytic and endophytic communities in this study should be considered operational rather than absolute. Both surface‐sterilized plant samples and soil samples were divided into two portions. The portion designated for microbial isolation underwent homogenization with sterile water through grinding and was subsequently plated onto Potato Dextrose Broth (PDB) medium, whereas the remaining portion reserved for genomic DNA extraction and polyphyllins analysis was maintained at −80°C. This study focused on characterizing bacterial communities in BS, RS, and the endosphere (root, stem, and leaf) of *P. polyphylla*. Epiphytic (surface‐associated) communities were not included in the sequencing analysis. Although epiphytic microbiota were known to contribute to plant performance, pathogen defense, and metabolite transformation, and may serve as reservoirs for endophytic colonization [41, 42], they were outside the scope of the present investigation. Therefore, the recruitment and filtering processes discussed in this manuscript are limited to the transition from soil to endophytic compartments and do not encompass potential interactions with or contributions from surface‐associated microbial communities.

### 2.2. Polyphyllin Measurement

Frozen *P. polyphylla* samples were dried in an oven at 60°C until a constant weight was achieved. The dried samples were ground into powder (smaller than 40 mesh) and extracted using a Soxhlet extractor according on pharmacopoeia of the People′s Republic of China (ChP) (V2015). A 0.500 g aliquot of the dried powder was homogenized in 50 mL of ethanol and refluxed at 80°C for 30 min. After filtration to remove residues, the volume was adjusted to 50 mL with ethanol for HPLC. HPLC analysis was conducted on a Shimadzu LC‐20 AD Series instrument, complemented by an SPD‐20A UV–Vis detector. The column used was an ACQUITY UPLCTM BEH C18 column (217 × 2.1 mm, 1.7 *μ*m), operated at 25°C. The mobile phase consisted of acetonitrile (A) and H_2_O (B). The gradient elution program was as follows: 0~15.0 min, 70% → 40% B; 15.0~16.0 min, 40% → 98% B; 16.0~18.0 min, and 98% → 98% B. The flow rate was set at 1.0 mL/min. Standard curves were generated using a series of standard concentration, and detection was performed at 203 nm.

### 2.3. DNA Extraction, Sequencing, and Sequence Analysis

Root, stem, and leaf fragments were ground in a sterile mortar and pestle with liquid nitrogen. Total genomic DNA was extracted from the plant samples using the OMEGA Plant DNA Kit, whereas the OMEGA Soil DNA Kits was employed for soil samples total genomic DNA extraction. Genome integrity was checked via 1.5% agarose gel electrophoresis, and quality and concentration were measured using a NanoDrop ND‐1000 spectrophotometer. To reduce host DNA interference, the microbial genome was amplified using primer pairs fM1 (5 ^′^‐CCGCGTGNRBGAHGAAGGYYYT‐3 ^′^) and rC5 (5 ^′^‐TAATCCTGTTTGCTCCCCAC‐3 ^′^) [43]. The amplicons were purified with a DNA Clean‐Up Kit and high‐throughput sequenced of the PCR products on the Illumina Miseq platform (Miseq PE250). For each batch of DNA extraction, blank extraction controls (no sample) were processed in parallel. For PCR amplification, no‐template controls (sterile water instead of DNA template) were included in each run. These negative controls consistently yielded no visible bands on 1.5% agarose gels and no detectable amplification products, confirming the absence of cross‐contamination during DNA extraction and PCR amplification.

The initial data underwent processing utilizing QIIME 2 (Version 2020.6). The procedures encompassed data quality filtering, clustering with the Ribosomal Database Project (RDP), sequence alignment, and analysis of community dissimilarities [44]. The taxonomic assignment of 16S representative sequences was carried out using the RDP classifier, based on the Greengene database (Version 13.5). Although SILVA and GTDB have become more widely used reference databases in recent years, Greengenes 13.5 remains a well‐curated and stable database for 16S rRNA gene taxonomic assignment. Comparative evaluations have shown that although SILVA offers broader coverage, Greengenes performs comparably at the genus level, which was the primary focus of our taxonomic analysis. We acknowledge that database choice may affect fine‐scale taxonomic assignments, but our genus‐level conclusions are expected to be robust to this selection. Operational taxonomic units (OTUs) tables, feature tables, and feature sequences were obtained. Furthermore, the raw data were submitted to the NCBI SRA database under the accession number PRJNA1240086.

### 2.4. Statistical Analysis

To analyze the bacterial communities in *P. polyphylla*, 16S rRNA gene sequences were resampled to 10,000 reads per sample to assess alpha and beta diversity. Observed OTU richness, Shannon diversity index, Simpson diversity index, and Chao1 richness estimator were calculated. Rarefaction curves were generated to evaluate sequencing depth adequacy, and Good′s coverage scores were calculated in QIIME 2 based on 10,000 iterations. To test the sensitivity of our conclusions to the rarefaction depth, we additionally recalculated alpha diversity at rarefaction depths of 8000 and 12,000 reads; the relative patterns among compartments and correlations with polyphyllin concentrations remained consistent across all depths. The relative abundance of microbes was used to determine community composition at the phylum and genus levels, and Venn diagram was created to identify shared and unique OTUs. R software was employed to calculate bacterial phylogenetic diversity, correlations between polyphyllin content and microbial abundance, and the relationship between polyphyllin content and alpha diversity in *P. polyphylla*. All correlation analyses were subjected to multiple comparison correction using the Benjamini‐Hochberg False Discovery Rate (FDR) method. Correlations with FDR‐adjusted *q* < 0.05 were considered statistically significant. Bacterial community structures among distinct compartments were analyzed using nonmetric multidimensional scaling (NMDS) with Bray–Curtis dissimilarity as the distance metric. Analysis of similarity (ANOSIM) was applied to test for significant differences in bacterial communities. To verify that the observed correlations between microbial communities and polyphyllins were independent of tissue niche, we performed sequential PERMANOVA (adonis2) with marginal term testing, which partitions variance by compartment, growth year, and polyphyllin concentrations. The sources of microbial in different *P. polyphylla* compartments were traced using fast expectation‐maximization microbial source tracking (FEAST). Redundancy analysis (RDA) was conducted to explore the impact of environmental factors on the bacterial community composition in *P. polyphylla*.

### 2.5. Spot Inoculation Experiment

Through microbial isolation and cultivation from surface‐sterilized roots, stems, and leaves of *P. polyphylla*, a large number of endophytic strains were obtained. After 72 h of culture on potato dextrose agar (PDA) medium at 30°C, colonies of strain P6 appeared circular, with entire margins, smooth and moist surfaces, raised, light yellow to off‐white in color, opaque, and viscous in texture, measuring approximately 2–5 mm in diameter. Photographs of colonial morphology (both front and reverse views) are provided in Figure S1. The full‐length 16S rRNA gene sequence of strain P6 was amplified and sequenced using universal bacterial primers (27F/1492R). BLASTn analysis against the NCBI 16S rRNA database showed that the sequence shared > 99% identity with members of the genus *Pseudomonas*, placing strain P6 within this genus. The genome sequence was compared with reference genomes of closely related *Pseudomonas* species using the ANI calculator (https://www.ezbiocloud.net/tools/ani). ANI values > 95%–96% are considered the gold standard for species delineation in prokaryotes. The ANI between strain P6 and the type strain of *Pseudomonas palleroniana* was 98.68%, which is well above the accepted species threshold. Both the 16S rRNA gene sequence and whole‐genome data consistently identified this strain as *P. palleroniana* (designated as *P. palleroniana* P6, hereafter referred to as P6) (Table S1). Based on multiple lines of evidence—including the relative abundance in community analysis, the proportion among isolated endophytic genera, and the correlation between microbiota and polyphyllin content across different plant compartments—*Pseudomonas* was selected for spot inoculation assays to evaluate the effects of gradient concentrations of polyphyllin I and VII on P6 growth. Polyphyllin I and VII were dissolved in methanol filtered through 0.22‐*μ*m sterile filters to prepare stock solutions of 1 mg/mL. These stock solutions were further diluted with methanol (similarly filtered) to final concentrations of 80, 40, 20, 10, and 5 mg/L. For experimental groups, 2 mL of each diluted polyphyllin I or VII solutions were added to 50 mL of sterilized PDA medium, whereas the blank control group received 2 mL of methanol. After thorough mixing, the 50 mL medium was equally poured into 3 sterile petri dishes. One *μ*L aliquot of P6 bacterial suspension (1 × 10^6^ CFU/mL) was spot‐inoculated at the center and along two perpendicular diameters (dividing the plate into four equal quadrants) of each plate. All plates were incubated at 30°C, and bacterial growth was monitored daily to assess colony development under varying polyphyllin types and concentrations. PDA was chosen as the culture medium because it supports vigorous growth of *P. palleroniana* P6 and ensures consistency with the isolation conditions used for this endophytic strain. All treatments were performed on the same medium, allowing direct comparison of polyphyllins effects under standardized nutritional conditions.

## 3. Results

### 3.1. Distribution of Polyphyllin Content in Different Compartments

**Figure 1:**
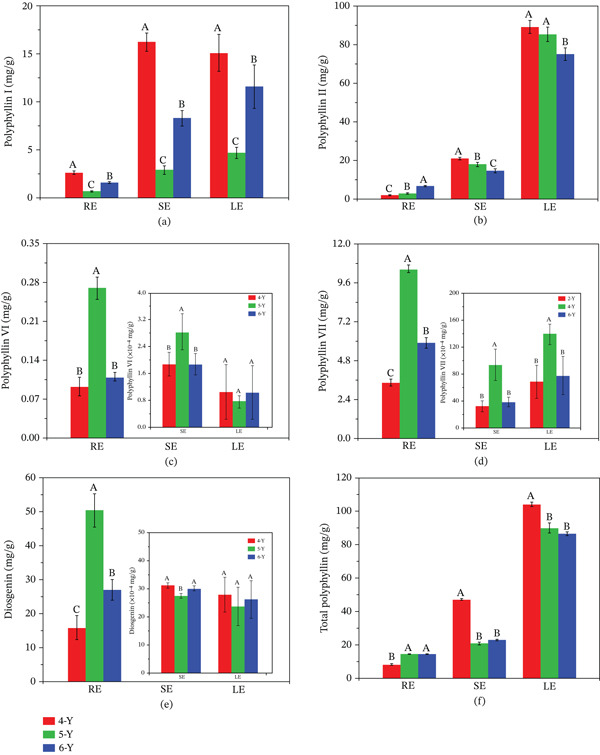
The content of Polyphyllin (a) I, (b) II, (c) VI, (d) VII, (e) diosgenin, and (f) total content of four polyphyllin in different compartments of *P. polyphylla* (mg/kg). 4‐Y, 5‐Y and 6‐Y represented *P. polyphylla* that were 4, 5, and 6 years, respectively. RE, SE, and LE represented root, stem, and leaf, respectively. The letters were generated based on pairwise comparisons using the Kruskal‐Wallis test followed by Dunn′s post hoc test (*p* < 0.05).

Significant variations in polyphyllin content were observed across different compartments and growth years of *P. polyphylla* (Figure 1). Notably, polyphyllin I was present in concentrations exceeding 1.00 mg/g in the roots, stems, and leaves. Polyphyllin II was particularly abundant, with its levels escalating progressively from roots to stems and culminating in the leaves, where it peaked at over 80 mg/g. The concentration of polyphyllin I and II was notably higher in the aerial parts, specifically the stems and leaves, compared to the roots. In contrast, polyphyllin VI was most concentrated in the roots, with levels dozens of times higher than in the stems and leaves, and all root samples contained over 0.07 mg/g of polyphyllin VI. However, its content decreased with increasing ecological niche spatial position. Similarly, polyphyllin VII was more prevalent in the roots than in the stems and leaves, with concentrations reaching over 10 mg/g at their highest and a minimum of 3.0 mg/g, displaying a trend of initial decline followed by an increase as the ecological niche spatial position advanced. Diosgenin content in the roots reached 10 mg/g, a magnitude hundreds of times greater than that in the stems and leaves. Statistical analysis of the total content of the four major polyphyllin revealed an ascending trend from roots to stems and leaves.

### 3.2. Bacterial Community Diversity in Different Compartments

A total of 1,119,315 high‐quality reads were obtained from 45 samples after quality control and host sequence exclusion. All sequences were clustered into 11,016 OTUs (97% similarity) after alignment with the 16S Greengene database. Venn diagram analysis of bacterial communities in different compartments of *P. polyphylla* revealed a decreasing trend in unique OTUs from BS to LE, with counts of 3,906, 735, 613, 251, and 185, respectively (Figure S2). Rarefaction curves reached saturation at approximately 5000–6000 reads (Figure S3a), and Good′s coverage estimates ranged from 94.5% to 98.6% across all samples (Figure S3b), confirming that sequencing depth was adequate for diversity characterization. The alpha diversity metrics (Shannon, Simpson, and Chao1) showed consistent trends across compartments (Figure S3c, S3d, S3e), with RS exhibiting the highest diversity, followed by RE, and then SE and LE.

**Figure 2:**
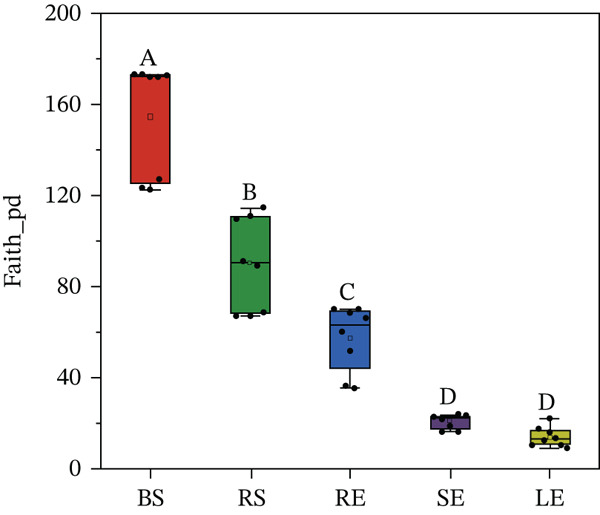
Phylogenetic diversity of bacterial communities in different compartments in the *P. polyphylla*.

**Figure 3:**
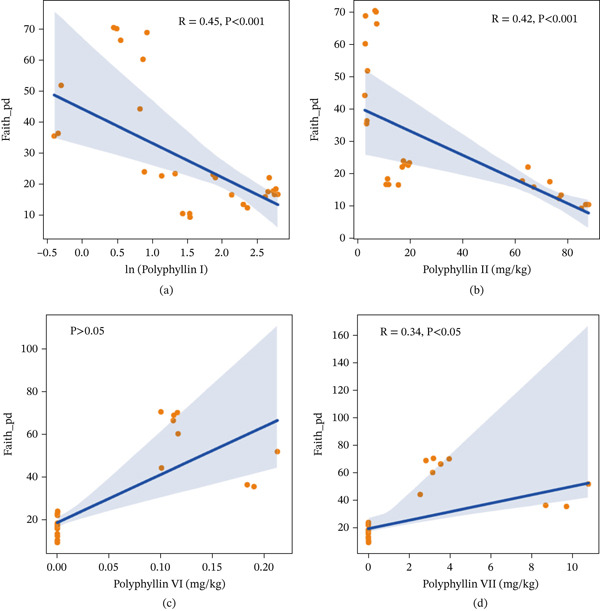
Linear regression plot of the relationship between polyphyllin and the *α*‐diversity of endophytes in *P. polyphylla*. (a) polyphyllin I, (b) polyphyllin II, (c) polyphyllin VI, (d) polyphyllin VII. *p* < 0.05 indicates that there is a significant difference (Benjamini‐Hochberg FDR‐corrected for multiple comparisons).

**Figure 4:**
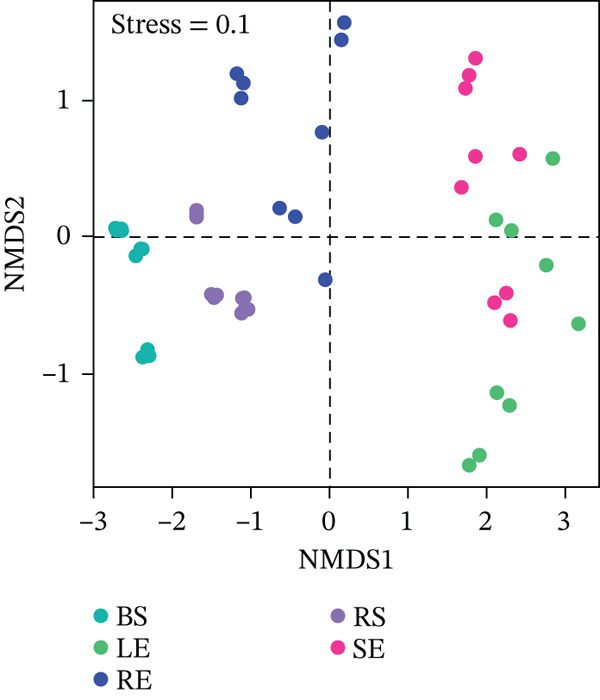
Nonmetric multidimensional scaling (NMDS) plots showing the effects of compartments on root‐associated bacterial community base on Bray‐curtis distance.

Phylogenetic diversity analysis (Figure 2) showed significant differences in the *α*‐diversity among these compartments, with soil bacterial communities exhibiting the highest *α*‐diversity. The *α*‐diversity of endophytic bacterial in *P. polyphylla* decreased with distance from the soil. Linear regression analysis (Figure 3) indicated that polyphyllin I and II were significantly negatively correlated with endophytic microbial *α*‐diversity, particularly polyphyllin II. In contrast, polyphyllin VII was significantly positively correlated, whereas polyphyllin VI showed not significant correlation. NMDS using Bray–Curtis distances showed clear separation of bacterial communities in different *P. polyphylla* compartments, with similar samples clustering together (Figure 4). ANOSIM confirmed significant differences in bacterial community structure among compartments (*R* ^2^ = 0.845, *p* = 0.001), with niche differentiation having a much greater impact than planting years (*R* ^2^ = 0.041, *p* = 0.001) (Table S2). Niche differentiation accounted for 84.5% of the variation in bacterial communities, whereas planting years explained only 4.1%. In samples from different years, niche effects on community structure were significant, explaining over 86.0% of the variance (*p* ≤ 0.001). For bacterial communities, the year′s variance explained exceeded 95.0% (*p* ≤ 0.001), whereas for soil communities, it was 81.6% and 68.4%, respectively. The PERMANOVA results (Table S3) confirmed that polyphyllin content remained a significant factor structuring bacterial communities after statistically controlling for compartment and year (*D* *F* = 2, *F* = 2.08, *p* = 0.001). This indicates that the observed microbiota–metabolite associations were not merely confounded by tissue identity but represent genuine biological relationships.

### 3.3. Bacterial Community Composition in Different Compartments

**Figure 5:**
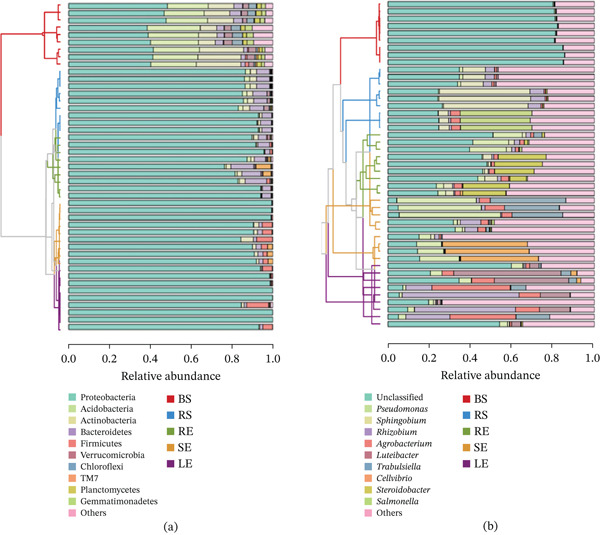
Microbial composition in different compartments of the *P. polyphylla* at (a) phylum level and (b) genus level.

The 11016 OTUs (97% similarity) belong to 49 phyla and 509 genera. At the phyla level, the composition of bacterial communities in different compartments of *P. polyphylla* showed significant differences, and the phylogenetic distance of bacterial communities in samples from the same compartment was closer (Figure 5a). Proteobacteria dominated the microbial composition in all samples except RSs, with relative abundance exceeding 80%. In the RS, the average relative abundances were 41.35% for Proteobacteria, 24.47% for Actinobacteria, and 18.78% for Firmicutes. The roots and RS have a more similar community composition structure, with the higher relative abundance of Bacteroidetes. The dominant phylum in stems and leaves is Firmicutes. There are also significant differences in the composition of bacterial communities among different compartments of *P. polyphylla* on the genus level (Figure 5b). The vast majority of microbes in the RSs were unclassified. The dominant genera in RSs were *Sphingomonas* (19.6%), *Salmonella* (10%), *Rhizobia* (4.0%), and *Agrobacterium* (2.1%). In the roots, stems, and leaves of *P. polyphylla*, the predominant microbial genera are *Pseudomonas*, *Sphingomonas*, *Agrobacterium*, *Rhizobium*, *Steroidobacter*, *Trabulsiella*, and *Luteibacter*.

**Figure 6:**
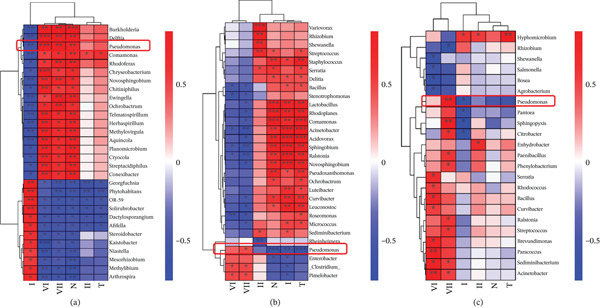
Correlation heat map of endophytic microbes and saponins in different compartments of the *P. polyphylla*. (a) In root (RE), (b) In stem (SE), and (c) In leaf (LE). VII, VI, II, I, N and T represent polyphyllin VII, VI, II, I, diosgenin, and total four polyphyllin, respectively. Significant differences indicated by asterisks: ∗, *p* < 0.05; ∗∗, *p* < 0.01; ∗∗∗, *p* < 0.001 (Benjamini‐Hochberg FDR‐corrected for multiple comparisons).

Due to significant differences in the composition of endophytic bacterial communities and polyphyllin content in the roots, stems, and leaves of *P. polyphylla*. We further analyzed the correlation between endophytic bacteria (genus level) and polyphyllin content (Figure 6). The results indicate that there were differences in species with strong correlation with polyphyllin content in different compartments. *Burkholderia*, *Delftia*, and *Pseudomonas* exhibited the most substantial correlation with the levels of polyphyllin in the roots. Specifically, these bacteria showed a significant negative correlation with polyphyllin I and a positive correlation with polyphyllin VI, VII, and diosgenin. In stems, *Pseudomonas* was significantly negatively correlated with polyphyllin I, diosgenin and total four polyphyllin, while significantly positively correlated with polyphyllin VII. Besides, the correlation between multiple genera and polyphyllin such as *Sphingomonas*, *Novosphingobium*, and *Rhodoplanes* was opposite to that of *Pseudomonas*. Within the leaves, the presence of *Pseudomonas*, *Citrobacter*, and *Pantoea* displayed a significant negative correlation with the levels of polyphyllin I, while they exhibited a positive correlation with polyphyllin VII.

### 3.4. The Influencing Factors of Bacterial Communities in Different Compartments

**Figure 7:**
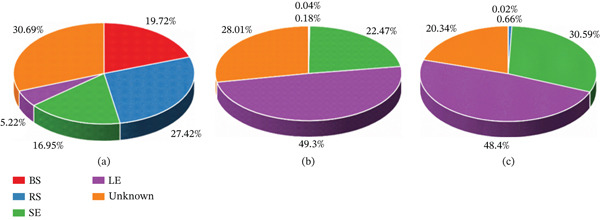
Fast expectation‐maximization microbial source tracking (FEAST) showing the source of microbes in different compartments of the *P. polyphylla*. (a) source tracking of RE, (b) source tracking of SE, and (c) source tracking of LE.

The FEAST method was used to track the microbial sources in various compartments of *P. polyphylla* (Figure 7). FEAST analysis revealed differences in the inferred source contributions among compartments based on compositional similarity. The largest contribution of microbes in the roots is RS (27.42%), followed by BS (19.72%), SE (16.95%) and LE (5.22%). The main contribution of microbes in the stem is LE (49.30%) and RE (22.47%), whereas the proportion of microbes from the BS (0.04%) and RS (0.18%) is negligible. The main contribution of microbes in the leaf is the SE (48.40%) and RE (30.59%). However, there are microbes of unknown origin in the roots, stems, and leaves, accounting for 30.69%, 28.01%, and 20.34%, respectively.

**Figure 8:**
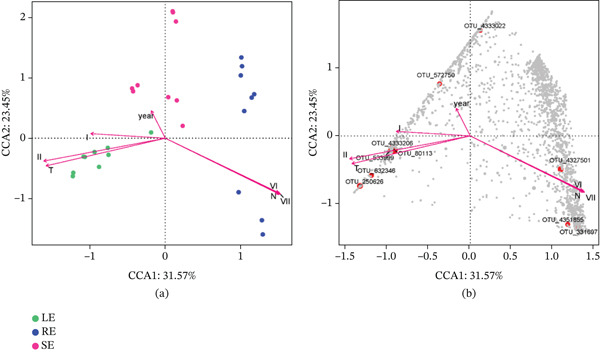
Redundancy analysis (RDA) of the correlation between polyphyllin and the composition of the bacterial communities.

RDA was performed to analyze the correlation between bacterial community OTU and various environmental factors (Figure 8a). The results showed that polyphyllin VI, VII, and diosgenin had the greatest impact on the bacterial community of *P. polyphylla*, followed by polyphyllin II and I. The impact of environmental factors on the OTUs of endophytic microbes in *P. polyphylla* indicates that the majority of OTUs were distributed in the second and fourth quadrants and were positively correlated with polyphyllin VI, VII, and diosgenin. A small portion of OTUs were distributed in the first and third quadrants and were positively correlated with the sum of polyphyllin I, II, and four types of polyphyllin (Figure 8b). As shown in Table S4, OTUs (OTU_4351855, OUT_4327501, and OUT_331697) with higher relative abundance and positive correlation with polyphyllin VI, VII, and diosgenin were all the Proteobacteria. Among them, OTU_4351855 and OTU_4327501 belong to *Pseudomonas*, and OUT_331697 belongs to *Enterobacterium*. OTUs (OTU_250626 OTU_632346, OTU_80113, OTU_4394926, OTU_4333206, OTU_533999, and OTU_572750) with higher relative abundance and positive correlation with the sum of polyphyllin I, II, and the four polyphyllin were also Proteobacteria. OTU_572750, OTU_4394926, and OTU_632346 belong to *Enteractor*, OTU_250626 and OTU_533999 belong to *Luteiactor*, OTU_80113 and OTU_4333206 were the *Rhizobium* and *Agrobacterium* of Rhizobiaceae, respectively.

### 3.5. Effects of Varying Polyphyllin Concentrations on *Pseudomonas* sp. Growth

**Figure 9:**
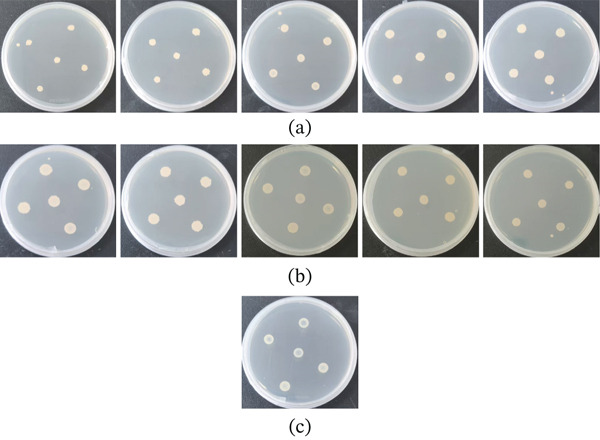
Effects of varying polyphyllin concentrations on *Pseudomonas* sp. growth. The result of *Pseudomonas* growing on culture medium for 7 days. (a) From left to right, 2 mL of polyphyllin I with concentrations of 80, 40, 20, 10, and 5 mg/L were added in sequence. (b) From left to right, 2 mL of polyphyllin VII with concentrations of 80, 40, 20, 10, and 5 mg/L were added in sequence. (c) Added equal volume of methanol.

The growth of P6 on the medium was continuously observed for 7 days. There was almost no difference in the growth of P6 in the three parallel experiments, so one group was selected for display. On the medium containing polyphyllin I, the growth rate gradually slows down with the increase of pariphyllin I content (Figure 9a). The increase in the content of pariphyllin I is not conducive to the growth of P6 and has an antagonistic relationship. On the medium containing pariphyllin VII, as the content of pariphyllin VII increases, the growth rate gradually accelerates (Figure 9b). High content of pariphyllin VII is beneficial to the growth of P6. The growth of the control group was similar to that of the group treated with 2 mL of 5 mg/L of pariphyllin I or pariphyllin VII (Figure 9c). The results of the point inoculation experiment once again confirmed the conclusion that pariphyllin I is negatively correlated with P6 and positively correlated with pariphyllin VII. Maybe, P6 have a synergistic effect on the synthesis of pariphyllin VII. This may also one of the important reasons why P6 is abundant in the roots of *P. polyphylla* and shows a downward trend from roots, stems, and leaves. However, whether strain P6 has a direct impact on the synthesis of pariphyllin VII still needs in vitro experimental verification.

## 4. Discussion

### 4.1. Polyphyllin Distribution Pattern in Different Compartments of *P. polyphylla*

Over an extended period, polyphyllin have been regarded as the key active components within the medicinal materials of *P. polyphylla*. Understanding their distribution across various tissues is crucial for the medicinal application of *P. polyphylla* [45]. The phenomenon that different tissues of plants have varying secondary metabolites is common [46, 47]. In this study, it was found that there are very noticeable differences in the types and contents of polyphyllin in different compartments of *P. polyphylla* (Figure 1). Studies on *P. polyphylla* showed that polyphyllin II is only present in the leaves during the fruiting stage, while the roots contain polyphyllin I, II, and VII [48]. The leaves are considered to be the primary site for the synthesis of polyphyllin, with genes related to the polyphyllin synthesis pathway being highly expressed in *P. polyphylla*′s leaves [49]. This is consistent with our experimental results, which show that polyphyllin, particularly polyphyllin I and II, are most content in the leaves. The study indicates that polyphyllin I and II are formed by attachment of the glycosylation of diosgenin at the 3‐position, whereas polyphyllin VI and VII are derivatives of protopanaxatriol [12]. Large amounts of polyphyllin I and II were detected in the leaves, but diosgenin was not found, whereas a higher content of diosgenin was detected in the roots. Thus, we speculate that the majority of diosgenin in *P. polyphylla*′s leaves and stems undergoes glycosylation to form polyphyllin I and II, whereas diosgenin in the roots is not all converted, resulting in a higher residual amount of diosgenin. According to the Chinese pharmacopoeia (V2020), for the roots of *P. polyphylla* used as medicine, the combined content of four types of polyphyllin should be no less than 0.6% [50]. In our study, even the lowest total polyphyllin content in the roots surpassed this threshold. Thus, all the samples could be regarded as high‐quality medicinal materials meet the requirements. Notably, the leaves of *P. polyphylla* had the highest total content of the four polyphyllin, followed by the stems, with both exceeding 20 mg/g. Although the pharmacopoeia records that solely the roots of *P. polyphylla* are utilized medicinally, mainly because the perennial roots have larger biomass and higher total cumulative polyphyllin content. Our research, consistent with previous studies [49, 51] indicates that the stems and leaves of *P. polyphylla* have the potential to be developed as a novel source of polyphyllin. Furthermore, the root of *P. polyphylla* contain relatively high levels of diosgenin, and if a higher conversion rate from diosgenin to polyphyllin I and II in the roots can be achieved, it would effectively enhance the content of medicinal ingredient and medicinal value of *P. polyphylla*.

### 4.2. Niche Differentiation and Polyphyllin Coregulate the Microbiome of *P. polyphylla*

Both ecological niche differentiation and plant secondary metabolites have been recognized as key drivers shaping plant‐associated microbial communities. Microorganisms exert pivotal influences on plant growth and phytochemical synthesis [52]. Deciphering the compartment‐specific microbial assembly mechanisms in plants is therefore critical for elucidating host‐driven microbial recruitment strategies with implications in agricultural biotechnology [53–55]. Epiphytic communities, which are surface related microbial communities, can affect plant health and secondary metabolism, and may serve as intermediates or reservoirs for endophytic colonization [41, 42]. Integrate the epiphytic and endophytic communities within the entire soil plant continuum, enables better delineation of the inferred scope of microbial recruitment and assembly in *P. polyphylla*. Although the distinction between epiphytic and endophytic communities in this study should be considered actionable, the consistent compartment specific patterns observed in multiple independent samples and years strongly support biological validity. In this study, we observed that niche differentiation and polyphyllin content jointly contribute to the distinct bacterial community structures across different compartments of *P. polyphylla*. The OTU Venn diagram and phylogenetic diversity results indicate that the BSs of *P. polyphylla* have the highest microbial richness and diversity, followed by the RSs. The species richness of endophytic bacteria in *P. polyphylla* tissue is significantly lower than that in root‐associated soil, and decreases with increasing distance from the soil. It is reasonable that BSs, as the most extensive and freely available compartment, harbor the highest species richness [22]. The BSs microbial communities are generally considered to be a subset of the RSs community [19]. For plants, in addition to inheriting microorganisms from the seeds, the REs mainly come from the RSs [56], and the plant roots also play a role in filtration [55, 57]. A total of 1235 OTUs were shared among BSs, RSs, and RE, indicating potential recruitment of these microorganisms from soil by the plant. This pattern aligns with previous studies demonstrating that soil serves as a primary microbial reservoir for plant roots [58, 59]. The observed lower *α*‐diversity observed in REs compared to RSs is consistent with findings from multiple plant species, including *Arabidopsis* [60], *maize* [19], and *Populus* [61], suggesting that this pattern may be a general feature of plant root microbiomes. Source tracking analysis of endophytic microbes in the *P. polyphylla* roots further confirmed that the root‐associated microbes are mainly contributed by RSs (27.42%) and the BSs (19.72%). The microbes in the stems of *P. polyphylla* are primarily contribute to the leaves and roots, whereas the microbes in the leaves contribute to the stems and roots, indicating mutual transmission of microorganisms in plant tissues [62]. These findings indicates that niche differentiation is a primary factor contributing to the differences in microbial diversity and species richness among the compartments of *P. polyphylla*; a conclusion that corroborates previous research findings [63]. It is important to note that although present study traced the origins of endophytic bacteria to soil compartments, the sources of the soil microbial communities themselves (e.g., air deposition and seed transmission) were not investigated. A comprehensive understanding of the full assembly trajectory along the soil‐plant continuum will require future studies that integrate atmospheric and seed‐borne microbial pools as potential sources. Meanwhile, FEAST infers potential source contributions based on compositional similarity between communities rather than directly demonstrating physical movement of microorganisms. Linear regression models were performed to assess the associations between polyphyllin concentrations and *α*‐diversity of bacterial communities in different compartments of *P. polyphylla*. The results indicated significant variations in the *α*‐diversity of bacterial communities for polyphyllin I, II, and VII. This fully demonstrates that, in addition to niche differentiation, the *α*‐diversity of bacterial communities among the compartments of *P. polyphylla* is also influenced by polyphyllin. Polyphyllin, known for their anti‐inflammatory and antimicrobial properties in traditional Chinese medicine, can inhibit the growth of various bacteria and viruses [33, 41]. Consequently, polyphyllin may affect the richness of microbes in various compartments of *P. polyphylla* by inhibiting the growth of certain microbes, thereby affecting their alpha diversity.

### 4.3. Polyphyllin‐Mediated Shaping of Endophytic Communities

In our study, the dominant bacterial genera in the rhizosphere and root of *P. polyphylla* are *Pseudomonas*, *Sphingomonas*, *Rhizobium*, and *Agrobacterium*. These microbial genera are also listed as the most common microorganisms in the roots and rhizosphere of plants [3, 42]. They are capable of fixing nitrogen, promoting root formation, and are extremely beneficial for host growth and development [64]. Previous studies have shown that *Sphingobium* might promote metabolism and energy conversion [65]. *Rhizobium* was thought to participate in the production of steroidal saponins [66]. *Rhizobium* and *Spirochetes* are considered the core bacterial genera of wheat and can promote its growth [67]. The core bacterial taxa selectively enriched in the rhizosphere and roots of *Salvia miltiorrhiza* to promote plant growth are *Pseudomonas*, *Sphingomonas*, and *Pantoea* [68]. The dominant genera of endophytic bacteria in *P. polyphylla* stems are mainly *Pseudomonas*, *Fibrobacter*, and *Trabulsilla*, whereas the leaves are mainly occupied by *Pseudomonas*, *Agrobacterium*, and *Trabulsilla*. *Fibrovibrio* and *Trabulsilla* are known to possess cellulose and lignin degradation abilities [10], and their presence in stems and leaves—tissues rich in these compounds—suggests a possible functional adaptation to these microhabitats. *Pseudomonas* is a dominant species in each compartment of *P. polyphylla*, indicating that it may play an important role in the growth and metabolism of *P. polyphylla*. Previous research has shown that the leaves extract of *P. polyphylla* has an inhibitory effect on the growth of *Pseudomonas* [41], suggesting that polyphyllin I in the leaf extract should play an important role in this process. The point connection experiment confirmed the conclusion that high content of polyphyllin I can inhibit the growth of *Pseudomona*. In our studies, a significant negative correlation was found between polyphyllin I and microbes belonging to *Pseudomonas* in all three compartments of the roots, stems, and leaves of *P. polyphylla*. The content of polyphyllin I is negatively correlated with the *α*‐diversity of endophytic microbes, with the highest *α*‐diversity observed in the roots, consequently resulting in the lowest content of polyphyllin I. In the roots, stems, and leaves of *P. polyphylla* at the 4th, 5th, and 6th years of growth, the relative abundance of *Pseudomonas* observed a decreasing and then increasing trend, respectively, while the content of polyphyllin I also showed the same trend. It is inferred that an increase in the abundance of *Pseudomonas* will promote the transformation of diosgenin into polyphyllin I, leading to a certain inhibitory effect on *Pseudomonas* through the production of a large amount of polyphyllin I. There appears to be a negative correlation between polyphyllin I content and *Pseudomonas* abundance in *P. polyphylla* tissues, suggesting a possible reciprocal interaction that warrants further experimental investigation. Furthermore, it also inspires us to adjust the abundance of *Pseudomonas* in the soil during the cultivation of *P. polyphylla* according to practical needs, thereby regulating the content of various polyphyllins in different compartments. However, the colonization ability, metabolic transformation capacity, and effects on polyphyllin biosynthesis in *P. polyphylla* of P6 remain to be further explored.

## 5. Conclusion

The microbial differentiation across compartments of *P. polyphylla* is largely attributed to ecological niches differentiation, whereas the endophytic bacterial community also shows a certain correlation with polyphyllins content. In addition, the richness of microbial species in the root system (including root circumference, rhizosphere, and inner root) is higher, making it an important area for studying the interaction between *P. polyphylla* and microbes. These findings significantly enhance the understanding of bacterial communities and polyphyllin synthesis in various ecological niches of *P. polyphylla*. *Pseudomonas* is a dominant genus across compartments of *P. polyphylla* and shows significant correlations with polyphyllin content. These correlative patterns, together with the in vitro growth responses of a representative strain to purified polyphyllins, suggest a potential interaction that merits further investigation into the functional roles of this genus in polyphyllin‐associated processes. This study focuses on the composition and recruitment mechanism of bacterial communities in different compartments of *P. polyphylla*, analyzes the ecological niche distribution pattern and influencing factors of bacterial communities in *P. polyphylla*, and provides theoretical reference for the rational planting and utilization of traditional Chinese medicine *P. polyphylla*.

## Author Contributions

X.L. conceived and supervised the project. Y.L., S.F., X.W., and S.L. designed this study. S.F. and S.L. performed the most of experiments. X.W., Y.D., K.Z., Z.D., and J.Z. assisted with performance of the experiment. X.W. visualized the analysis and wrote the manuscript. L.J. and N.I. polished the language of the article. Y.L. and S.F. revised the article.

## Funding

This study was supported by the National Natural Science Foundation of China, 10.13039/501100001809, 31570113.

## Disclosure

All authors have read and approved the manuscript.

## Conflicts of Interest

The authors declare no conflicts of interest.

## Supporting information


**Supporting Information** Additional supporting information can be found online in the Supporting Information section. Figure S1 Front (a) and reverse (b) views of the colonial morphology of strain *Pseudomonas palleroniana* P6 on PDA plate medium. Supporting Information Figure S2 Venn diagram of microbial richness in different compartments of the *P. polyphylla* at OTU level. Supporting Information Figure S3 Alpha diversity assessment. (a) Rarefaction curves showing observed OTU richness as a function of sequencing depth for all samples. (b) Rarefaction curves showing Good′s coverage estimates across all samples. (c) Rarefaction curves showing the Shannon diversity index across all samples. (d) Rarefaction curves showing the Simpson diversity index across all samples. (e) Rarefaction curves showing the Chao1 richness estimator across all samples. Abbreviaions: BS, bulk soil; RS, rhizosphere soil; RE, root endosphere; SE, stem endosphere; and LE, leaf endosphere. Supporting Information Table S1 Results of identification of strain P6. Supporting Information Table S2 ANOSIM statistical analyses testing the effects of the compartments and planting years on the bacterial community. Supporting Information Table S3 Sequential PERMANOVA (adonis2) marginal term test partitioning variance explained by compartment, growth year, and polyphyllin concentrations. Supporting Information Table S4 Classification of polyphyllin‐related OTUs.

## Data Availability

The data that support the findings of this study are openly available in NCBI at https://www.ncbi.nlm.nih.gov/bioproject/?term=PRJNA1240086, reference number PRJNA1240086.
