# Supplementary material for: Compartment‐Specific Variation in Bacterial Microbiome and Polyphyllin Profiles in Paris polyphylla
Source: Int J Microbiol. 2026 Jul 8;2026:1725012. doi: 10.1155/ijm/1725012 (PMC13342703; doi:10.1155/ijm/1725012)
Supplement: Supplementary file 1 — Supporting Information Additional supporting information can be found online in the Supporting Information section. Figure S1 Front (a) and reverse (b) views of the colonial morphology of strain Pseudomonas palleroniana P6 on PDA plate medium. Supporting Information Figure S2 Venn diagram of microbial richness in different compartments of the P. polyphylla at OTU level. Supporting Information Figure S3 Alpha diversity assessment. (a) Rarefaction curves showing observed OTU richness as a function of sequencing depth for all samples. (b) Rarefaction curves showing Good′s coverage estimates across all samples. (c) Rarefaction curves showing the Shannon diversity index across all samples. (d) Rarefaction curves showing the Simpson diversity index across all samples. (e) Rarefaction curves showing the Chao1 richness estimator across all samples. Abbreviaions: BS, bulk soil; RS, rhizosphere soil; RE, root endosphere; SE, stem endosphere; and LE, leaf endosphere. Supporting Information Table S1 Results of identification of strain P6. Supporting Information Table S2 ANOSIM statistical analyses testing the effects of the compartments and planting years on the bacterial community. Supporting Information Table S3 Sequential PERMANOVA (adonis2) marginal term test partitioning variance explained by compartment, growth year, and polyphyllin concentrations. Supporting Information Table S4 Classification of polyphyllin‐related OTUs. [file IJM-2026-1725012-s001.docx]

**Compartment-Specific Variation in Bacterial Microbiome and Polyphyllin Profiles in *Paris polyphylla***

Xinhong Wu^1^, Yan Deng^2^, Shihui Li^1^, Kai Zou^3^, Zhenchun Duan^1^, Nazidi Ibrahim^1^, Jin Zhou^1^, Luhua Jiang^1^, Xueduan Liu^1^, Shaodong Fu^1*^, Yili Liang^1*^

^1^ School of Resource Processing and Bioengineering, Key Laboratory of Biometallurgy of Ministry of Education, Central South University, Changsha 410083, China

^2^ Hunan Institute of Microbiology, Hunan Academy of Agricultural Sciences, Changsha 410125, China

^3^ College of Advanced Materials Engineering, Jiaxing Nanhu University, Jiaxing, Zhejiang, China

* Corresponding authors.

E-mail addresses: fsd1032725536@gmail.com (Shaodong Fu), liangyili6@csu.edu.cn (Yili Liang)

**Figures**


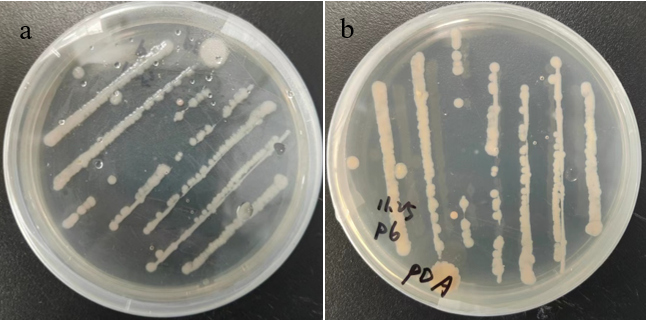


Figure S1 Front (a) and reverse (b) views of the colonial morphology of strain *Pseudomonas palleroniana* P6 on PDA plate medium.


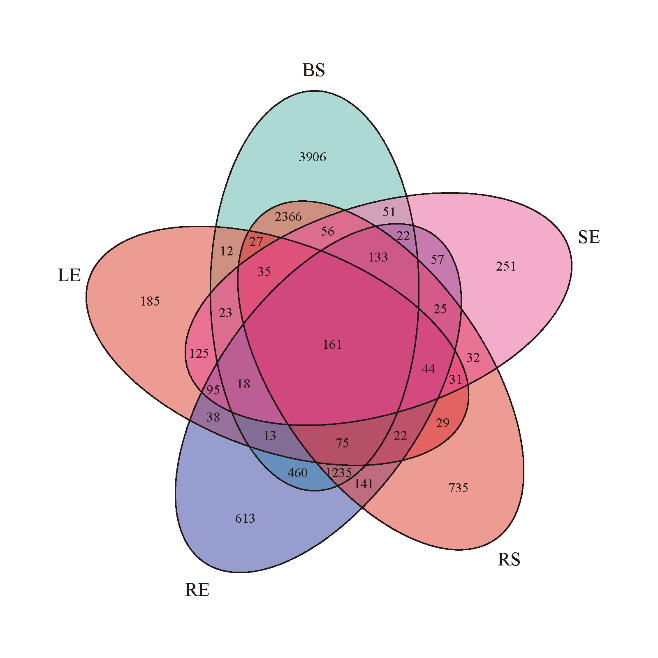


Figure S2 Venn diagram of microbial richness in different compartments of the *P. polyphylla* at OTU level.


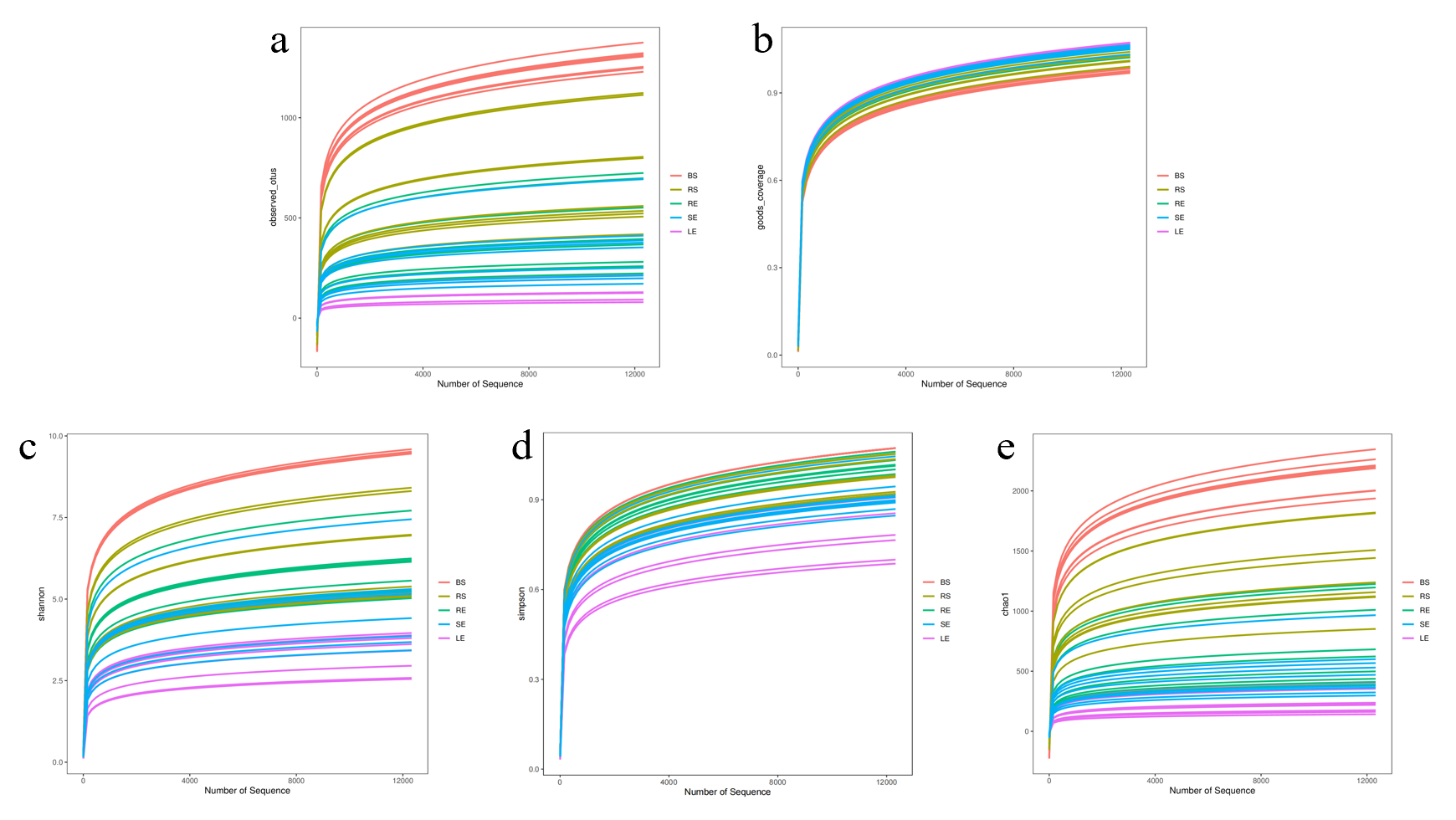


Figure S3 Alpha diversity assessment. (a) Rarefaction curves showing observed OTU richness as a function of sequencing depth for all samples. (b) Rarefaction curves showing Good's coverage estimates across all samples. (c) Rarefaction curves showing the Shannon diversity index across all samples. (d) Rarefaction curves showing the Simpson diversity index across all samples. (e) Rarefaction curves showing the Chao1 richness estimator across all samples. BS: bulk soil; RS: rhizosphere soil; RE: root endosphere; SE: stem endosphere; LE: leaf endosphere.

**Tables**

Table S1 Identification results of strain P6.

| Reference strain | 16S rRNA gene sequence identity (%) | ANI value (%) |
| --- | --- | --- |
| *Pseudomonas palleroniana* strain MAB3 | 100% | 98.68% |

Table S2: ANOSIM statistical analyses testing the effects of the compartments and planting years on the bacterial community.

| Samples | Variables | R^2^ | *p* |
| --- | --- | --- | --- |
| All samples (n = 45) | Compartment | 0.845 | 0.001 |
|  | Planting years | 0.041 | 0.001 |
| LE (n = 9) | Planting years | 0.954 | 0.001 |
| SE (n = 9) | Planting years | 0.963 | 0.001 |
| RE (n = 9) | Planting years | 0.986 | 0.001 |
| RS (n = 9) | Planting years | 0.816 | 0.001 |
| BS (n = 9) | Planting years | 0.684 | 0 |
| Y4 (n = 15) | Compartment | 0.860 | 0.001 |
| Y5 (n = 15) | Compartment | 0.943 | <0.001 |
| Y6 (n = 15) | Compartment | 0.898 | <0.001 |

Table S3 Sequential PERMANOVA (adonis2) marginal term test partitioning variance explained by compartment, growth year, and polyphyllin concentrations.

|  | Df | Variance | F | Pr (>F) |
| --- | --- | --- | --- | --- |
| Model | 2 | 49.2865269744872 | 2.07731062885404 | 0.001 |
| Residual | 24 | 284.713473025513 |  |  |

Table S4 Classification of polyphyllin-related OTUs.

| Feature | Phylum | Family | Genus |
| --- | --- | --- | --- |
| OUT_4351855 | Proteobacteria | Pseudomonadaceae | *Pseudomonas* |
| OTU_4327501 | Proteobacteria | Pseudomonadaceae | *Pseudomonas* |
| OTU_331697 | Proteobacteria | Enterobacteriaceae | *Enterobacter* |
| OTU_4394926 | Proteobacteria | Enterobacteriaceae | *Enterobacter* |
| OTU_632346 | Proteobacteria | Enterobacteriaceae | *Enterobacter* |
| OTU_572750 | Proteobacteria | Enterobacteriaceae | *Enterobacter* |
| OTU_533999 | Proteobacteria | Xanthomonadaceae | *Luteibacter* |
| OTU_250626 | Proteobacteria | Xanthomonadaceae | *Luteibacter* |
| OTU_4333206 | Proteobacteria | Rhizobiaceae | *Agrobacterium* |
| OTU_80113 | Proteobacteria | Rhizobiaceae | *Rhizobium* |
